# Deliveries among teenage women – with emphasis on incidence and mode of delivery: a Swedish national survey from 1973 to 2010

**DOI:** 10.1186/1471-2393-13-204

**Published:** 2013-11-09

**Authors:** Rasmus Birch Tyrberg, Marie Blomberg, Preben Kjølhede

**Affiliations:** 1 Division of Obstetrics and Gynaecology, Department of Clinical and Experimental Medicine, Faculty of Health Sciences, Linköping University, S - 581 85 Linköping, Sweden; 2 Department of Obstetrics and Gynaecology, County Council of Östergötland, Linköping University, S - 581 85 Linköping, Sweden

**Keywords:** Adolescent birth, Incidence, Epidemiology, Mode of delivery, Neonatal outcome, Risk factors

## Abstract

**Background:**

Since the 1970-ies Sweden has actively developed strategies in social care, education and health care in order to counteract the negative consequences of adolescent parenthood. The aims of this study are to determine the annual incidence of singleton delivery among adolescents 1973–2010 and analyse obstetric and neonatal outcomes.

**Methods:**

A retrospective cohort study, using data from the Swedish Medical Birth Register was conducted. All singleton deliveries in Sweden between 1973 and 2010 were included. Totally 1,941,940 women had 3,761,576 deliveries during the period. Analyses of obstetric and neonatal outcome were restricted to 1992–2010. Adolescents were subdivided into three groups: <16 years (n = 472), 16–17 years (n = 5376), 18–19 years (n = 23560). The reference group consisted of women age 20–30 years (n = 893505). Data were analysed using multivariate logistic regression models adjusted for confounding factors and presented as crude and adjusted odds ratios with 95% confidence interval.

**Results:**

The annual incidence of teenage births decreased significantly from 7.7 to 1.6%. Teenagers were more likely to deliver normally vaginally (aOR 1.70 (95%CI 1.64-1.75), less likely to have Caesarean section (aOR 0.61 (95%CI 0.58-0.64), and had a greater risk of delivering prematurely (< 28 weeks)(aOR 1.61 (95%CI 1.31-2.00), but did not have more small-for-gestational-age babies (aOR 1.07 (95%CI 0.99-1.14). Risks of placenta previa, postpartum haemorrhage > 1000 ml and perineal rupture were significantly lower among teenagers. Although the rate with Apgar score <7 at 5 minutes was similar the teenager’s neonates showed less fetal distress and meconium aspiration.

**Conclusion:**

Adolescent births have steadily decreased in Sweden. Adolescents were more likely to be delivered vaginally than the adult women. The risks for obstetric maternal complications for adolescents were lower than for adult women except for the risk of prematurity.

## Background

Establishing a family as an adolescent has been associated with adverse neonatal outcome [1] and severe socioeconomic consequences [2]. Low maternal age has been shown to increase the risk of neonatal morbidity, premature birth, low birth weight, maternal anaemia [1,3-8] and post-gestational depression [9]. The obstetric care of adolescents seems to differ from that given to adult women since adolescents have a lower frequency of delivery through Caesarean section and instrumental vaginal delivery [4-6,10,11]. Although several studies have indicated that adolescents have significantly increased occurrence of adverse perinatal outcomes, such as prematurity, postpartum haemorrhage (PPH), preeclampsia, low birth weight/small for gestational age (SGA), fetal distress and fetal death, the results have not been in unanimous agreement [4,7,8,10-12].

Underlying reasons causing the differences in overall risks between adolescents and adult women have been attributed to poor socioeconomic family conditions [13] although this conclusion has been questioned [1]. Poor socioeconomic and vulnerable family conditions have in turn been seen as a factor leading to increased risk for giving birth as a teenager [1,2,14]. Regardless of socioeconomic background teenage mothers suffer from a higher mortality rate later in life than women who have given birth between 20–29 years of age [15].

Since the 1970-ies Sweden has actively developed strategies in social care, education and health care in order to counteract the negative consequences of adolescent parenthood. The most important strategy has been efforts to avoid adolescent pregnancies. This has been achieved by means of several different approaches including structured education programs in schools encouraging vocational training before family planning, a liberal view on adolescent sexuality, early introduction of youth health clinics and easy access to contraception and legislation providing for free legal abortion [16,17].

This paper reports on the changes in the incidence and ratio of adolescent births during the period 1973–2010 in Sweden and on an evaluation of obstetric and neonatal outcomes associated with giving birth as a teenager. The evolution of the incidence of adolescent birth rate in Sweden has not been reported on since the year 2000 [18]. Information about changes may be important for decision makers as well as for the health-care and social security systems. Furthermore an analysis of perinatal outcomes in the Swedish population will provide important knowledge of these changes that may be used to further improve the social, antenatal, obstetric and neonatal care for teenagers who become parents.

## Methods

This is a nationwide population-based cohort study of women with singleton pregnancies who gave birth in Sweden from January 1, 1973 through December 31, 2010. The data were extracted from the Swedish Medical Birth Register (MBR) [19]. The study was approved by the Regional Ethical Review Board in Linköping (Dnr 2011/479-31. Approved January 25; 2012), and by the board of the MBR.

The Swedish MBR was founded in 1973 and is administered by the Swedish National Board of Health and Welfare. It is compulsory for every health care provider to report to the register. Medical and other data on almost all (99%) deliveries in Sweden are listed in the register which also includes stillbirths after 22 weeks of gestation. It is based on copies of the standardized medical record forms completed at the maternity health care centres at the start of antenatal care, usually in gestational week 10–12, records from the delivery units, and the paediatric examination of the new-born. The system is identical throughout the country. A description and validation of the register content is available [19,20].

Women in the study population were grouped according to maternal age. The teenagers were subdivided into three groups: < 16 years; 16–17 years, and 18–19 years old. In the outcome analyses that were performed, all the teenagers as one group and the subgroups of teenagers were separately compared with a reference group of the women age 20–30 years.

The obstetric outcome variables studied were gestational age, mode of delivery, mode of onset of delivery, perineal rupture, preeclampsia, abruptio placentae, placenta previa, use of epidural analgesia and PPH > 1000 ml. The neonatal outcomes evaluated were small for gestational age (SGA) defined as birth weight more than 2 standard deviations (SD) below the mean birth weight adjusted for gestational age (sex and parity specific) according to a Swedish reference curve, large for gestational age (LGA) defined as birth weight more than 2 SD above the mean birth weight adjusted for gestational age (sex and parity specific), Apgar-score at 5 minutes, fetal distress (International Statistical Classification of Diseases and Related Health Problems – Ninth revision (ICD-9) codes 768.2-4; and ICD-10 codes P20.0, P20.1 and P20.9), aspiration of meconium, shoulder dystocia and stillbirth.

The list of available variables in MBR has been extended throughout the years that the register has been active. The obstetric and neonatal outcome data for the purpose of this study are those that have been available since 1992; therefore we limited the analyses of obstetric and neonatal outcomes to the years 1992 to 2010. From 1992 until June 2008 the MBR includes stillbirths after 28 weeks gestation and from July 2008 until 2010 all stillbirths after 22 weeks gestation are included. Each outcome studied was either marked in the Swedish Medical Birth Registry or registered according to the ICD.

Parity and the annual incidence of adolescent births are available since 1973 and are presented as absolute number of births and as the rate of adolescent births in the total population of women with singleton deliveries. In addition, we present the adolescent birth rate among the population of female teenagers. Information about the number of female teenagers in Sweden in the period 1973 to 2010 was extracted from Statistics Sweden [21], the government agency that produces official statistics about Sweden.

### Statistical analysis

The software STATISTICA 64 version 10 (StatSoft Inc. 2300 East 14^th^ St. Tulsa, OK 74104 USA) was used to carry out the statistical analyses. Data are presented as number and per cent or mean and one standard deviation (SD). Comparison of groups was accomplished by means of logistic regression analyses for categorical data and results are presented as odds ratios (OR) and 95% confidence intervals (CI). Continuous data were compared using analysis of covariance (ANCOVA). Multivariate models were used in order to adjust comparisons for the confounding factors. Maternal weight and length (enabled calculation of maternal Body Mass Index (BMI)), parity (1-3+), smoking habits in early pregnancy (unknown, no smoking, smoking) and year of delivery were thought to be potential confounding factors and were included as co-variates in the adjusted analyses. These variables were also extracted from the Swedish Medical Birth Register. The register information on these variables was obtained from the antenatal care centre records. Gestational age was added to the confounders in the analyses of Caesarean section (CS), preeclampsia and birth weight.

## Results

In the period 1973 – 2010, 1,941,940 women had 3,761,576 singleton deliveries as registered in the MBR. This dataset was used for the descriptive analysis of the annual incidence of adolescent births. Annually the birth rate varied between 80,000 and 120,000. The analyses of the obstetric and neonatal outcomes were restricted to the 922,913 singleton deliveries among women younger than 31 years that took place in the period 1992 – 2010.

### Incidence of adolescent birth 1973–2010

**Figure 1:**
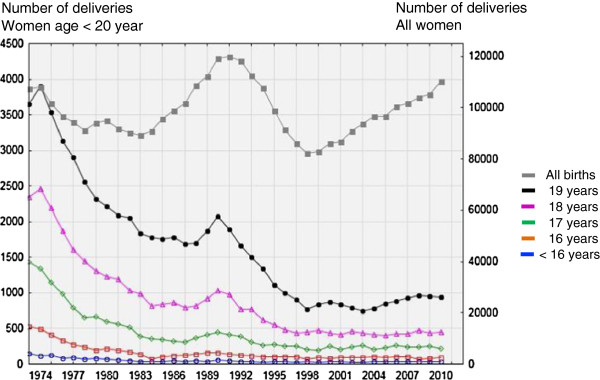
Annual incidence of singleton deliveries in Sweden between 1973 and 2010 in teenagers and the total population.

**Figure 2:**
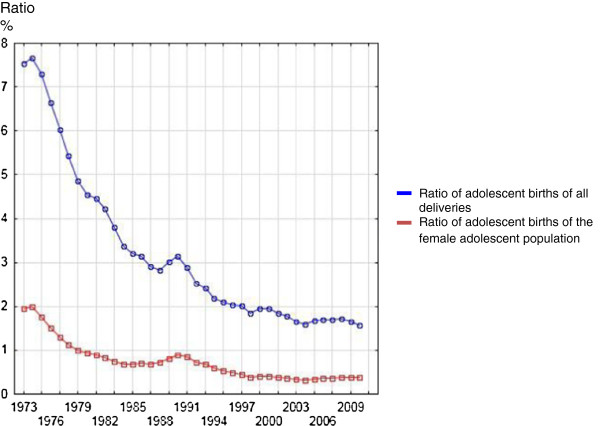
Ratios of adolescent births of all singleton deliveries and in all female adolescents.

The annual incidence of adolescent births subdivided according to the age of the adolescent and the total number of singleton deliveries are presented in Figure 1. The annual ratios of adolescent births of all singleton deliveries and of all adolescents are shown in Figure 2. The ratio of teenage births among all singleton births has steadily declined since 1974 from 7.7% to 1.6% in 2010. The ratio of adolescent births to total number of adolescents decreased in almost identical fashion from 2.0% to 0.4%.

**Figure 3:**
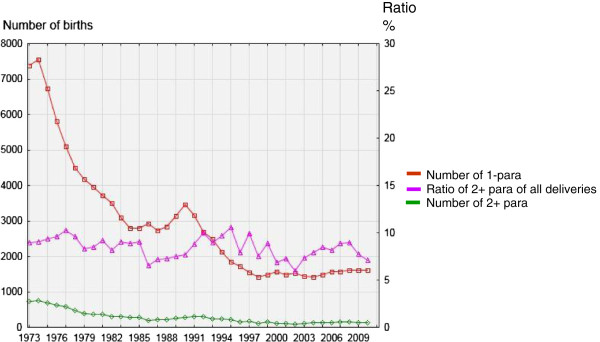
Annual number of 1-para and 2+ para teenagers, and ratios of teenagers 2 + para of all deliveries between 1973 and 2010.

The number of primiparous and multiparous adolescents declined significantly during the period but interestingly the proportion of teenagers who delivered more than once was rather constant at a level of 7-10% (Figure 3).

### Mode of delivery, obstetric and neonatal outcome 1992–2010

**Figure 4:**
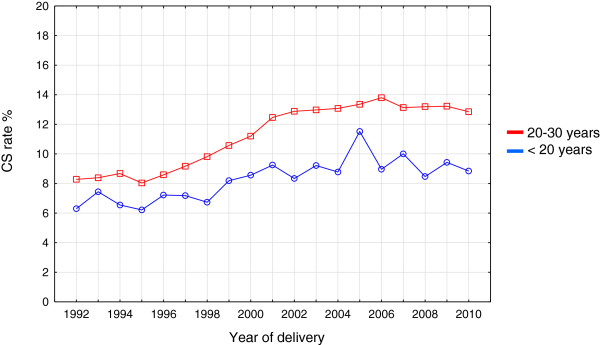
Annual Caesarean section (CS) rates in adolescents and women age 20–30 years between 1992 and 2010.

The demographic, obstetric and neonatal data of the study groups are depicted in Table 1, 2 and 3. The rate of delivery by CS was significantly higher in the adult women than in the teenager group. Irrespective of age the CS rate increased substantially in both groups but to a lesser degree in the teenage group (8.3 to 12.9% for adult women versus 6.3 to 8.8% for teenagers) as shown in Figure 4.

**Table 1:** Demographic characteristics of women with singleton births in the period 1992-2010

**Age groups**									
**Characteristic**		**< 16 years**		**16-17 years**		**18-19 years**		**20-30 years**	
		**(n=472)**		**(n=5376)**		**(n=23560)**		**(n=893505)**	
BMI (kg/m^2^)^†^		22.8	(3.4)	22.8	(3.8)	23.3	(4.2)	24.1	(4.3)
Smoking^†^		105	(22.7%)	1830	(34.7%)	7239	(31.3%)	114278	(12.8%))
Parity:	1	469	(99.4%)	5208	(96.9%)	21287	(90.4%)	471470	(52.8%)
	2	3	(0.6%)	163	(3.0%)	2131	(9.0%)	318522	(35.6%)
	3+	0	(0%)	5	(0.1%)	142	(0.6%)	103513	(11.6%)

Figures denote means and one SD or numbers and proportions.

BMI = body mass index.

^†^Reported length, weight and smoking habits at first antenatal visit.

**Table 2:** Descriptive obstetric characteristics of women with singleton births in the period 1992-2010

	**Age groups**							
**Characteristics**	**< 16 years**		**16-17 years**		**18-19 years**		**20-30 years**	
Spontaneous onset delivery	398	(84.3%)	4692	(87.3%)	20454	(86.8%)	753637	(84.3%)
Induced delivery	41	(8.7%)	434	(8.1%)	1985	(8.4%)	82566	(9.2%)
Normal vaginal delivery	396	(83.9%)	4599	(85.5%)	20006	(84.9%)	728163	(81.5%)
Forceps	2	(0.4%)	13	(0.2%)	96	(0.4%)	3812	(0.4%)
Vacuum extraction	29	(6.1%)	336	(6.3%)	1540	(6.5%)	63134	(7.1%)
CS^¥^	45	(9.5%)	429	(8.0%)	1916	(8.1%)	98390	(11.0%)
Elective CS^†^	20	(4.2%)	70	(1.3%)	316	(1.3%)	22105	(2.5%)
Acute CS^†^	13	(2.8%)	168	(3.1%)	686	(2.9%)	33265	(3.7%)
GA < 28 weeks	4	(0.9%)	25	(0.5%)	71	(0.3%)	1576	(0.2%)
GA < 32 weeks	7	(1.5%)	73	(1.4%)	207	(0.9%)	5447	(0.6%)
GA < 37 weeks	54	(11.5%)	384	(7.2%)	1411	(6.0%)	42891	(4.8%)
GA 37 – 41 weeks	387	(82.3%)	4620	(86.1%)	20622	(87.6%)	787840	(88.2%)
GA ≥ 42 weeks	29	(6.2%)	363	(6.8%)	1514	(6.4%)	62595	(7.0%)
Epidural analgesia*	175	(41.0%)	2099	(42.4%)	8658	(40.0%)	221265	(27.8%)
Perineal rupture grade 1-2*	68	(15.9%)	689	(13.9%)	3204	(14.8%)	177205	(22.3%)
Perineal rupture grade 3-4*	4	(0.9%)	55	(1.1%)	218	(1.0%)	14190	(1.8%)
Preeclampsia	12	(2.5%)	89	(1.7%)	415	(1.8%)	13980	(1.6%)
Abruptio placentae	1	(0.2%)	29	(0.5%)	113	(0.5%)	3317	(0.4%)
Placenta previa	2	(0.4%)	1	(0.0%)	14	(0.1%)	1501	(0.2%)
PPH > 1000 ml (VD)	10	(2.3%)	125	(2.5%)	523	(2.4%)	26305	(3.3%)
PPH > 1000 ml (CS)	0	(0%)	9	(2.1%)	18	(0.9%)	1200	(1.2%)

Figures denote numbers and proportions.

CS = Caesarean section; GA = gestational age at delivery; PPH = postpartum haemorrhage; VD = vaginal delivery.

¥ All CS independent of status of performance – acute or elective.

*Epidural analgesia and perineal rupture in vaginal deliveries.

^†^Caesarean section was subdivided into elective and acute CS from 1999.

**Table 3:** Descriptive neonatal outcome among women with singleton births in the period 1992-2010

	**Age groups**							
**Outcome**	**< 16 years**		**16-17 years**		**18-19 years**		**20-30 years**	
Birth weight (gram)	3300	(569)	3388	(574)	3414	(559)	3541	(551)
Adjusted birth weight (gram)^§^	3453	(20.0)	3476	(5.9)	3464	(2.9)	3483	(0.7)
Fetal distress	0	(0%)	15	(0.3%)	87	(0.4%)	3147	(0.4%)
Aspiration of meconium	0	(0%)	2	(0.0%)	24	(0.1%)	1287	(0.1%)
Shoulder dystocia	1	(0.2%)	14	(0.3%)	52	(0.2%)	3499	(0.4%)
Stillbirth	1	(0.2%)	22	(0.4%)	65	(0.3%)	2449	(0.3%)
SGA	19	(4.1%)	180	(3.4%)	842	(3.6%)	20284	(2.3%)
LGA	3	(0.6%)	89	(1.7%)	443	(1.9%)	28570	(3.2%)
Apgar score at 5 minutes < 7	8	(1.7%)	68	(1.3%)	280	(1.2%)	9280	(1.0%)

Figures denote means and one SD or numbers and proportions.

LGA = Large for gestational age; SGA = Small for gestational age.

§ Adjusted for the covariates BMI and smoking habits at first antenatal care visit, parity, gestational age at delivery and year of delivery at their means. Dispersion of the adjusted birth weight is presented as one standard error of the mean (SEM).

The results of the multivariate analyses models of obstetric and neonatal outcomes are presented in Table 4 and 5, respectively. Compared with adult women age 20–30 years the teenagers had a significantly higher likelihood of having a normal vaginal delivery and of giving birth prematurely. Similarly, the teenagers had epidural analgesia significantly more often in vaginal deliveries than did adult women. In contrast with this, the teenagers were delivered instrumentally (abdominally as well as vaginally) significantly less often, and the vaginal deliveries caused significantly fewer perineal ruptures and PPH > 1000 ml. Placenta previa was less likely to occur in the teenagers and the occurrence of abruptio placentae and preeclampsia was equal to that seen among the adult women. The results for the women < 16 years of age differed concerning spontaneous onset of labour, forceps, epidural analgesia, placenta previa, and PPH compared with the other adolescent groups.

**Table 4:** Mode of delivery and obstetric data among teenage women with singleton births in the period 1992–2010

	**Age groups**							
	**All teenagers**		**< 16 years**		**16-17 years**		**18-19 years**	
**Characteristics**	**Crude**	**Adjusted**^**†**^	**Crude**	**Adjusted**^**†**^	**Crude**	**Adjusted**^**†**^	**Crude**	**Adjusted**^**†**^
Spontaneous onset delivery	1.23 (1.19-1.27)	1.17 (1.13-1.21)	1.00 (0.78-1.28)	0.92 (0.72-1.18)	1.27 (1.17-1.38)	1.18 (1.09-1.28)	1.22 (1.17-1.27)	1.17 (1.13-1.22)
Induced delivery	0.90 (0.86-0.93)	0.92 (0.88-0.96)	0.93 (0.68-1.29)	0.95 (0.69-1.32)	0.86 (0.78-0.95)	0.91 (0.82-1.00)	0.90 (0.86-0.95)	0.92 (0.88-0.97)
Normal vaginal delivery	1.29 (1.25-1.33)	1.70 (1.64-1.75)	1.18 (0.92-1.51)	1.61 (1.26-2.06)	1.34 (1.25-1.45)	1.80 (1.66-1.94)	1.28 (1.23-1.33)	1.67 (1.61-1.74)
Forceps	0.88 (0.73-1.06)	0.56 (0.46-0.68)	0.99 (0.25-3.98)	0.67 (0.17-2.68)	0.57 (0.33-0.98)	0.34 (0.19-0.60)	0.95 (0.78-1.17)	0.61 (0.50-0.76)
Vacuum extraction	0.91 (0.87-0.95)	0.59 (0.56-0.62)	0.86 (0.59-1.25)	0.50 (0.34-0.74)	0.88 (0.78-0.98)	0.54 (0.49-0.61)	0.92 (0.87-0.97)	0.60 (0.57-0.63)
CS, all	0.71 (0.69-0.75)	0.61 (0.58-0.64)	0.85 (0.63-1.16)	0.65 (0.47-0.88)	0.70 (0.63-0.77)	0.58 (0.52-0.64)	0.72 (0.68-0.75)	0.61 (0.58-0.64)
CS elective 1999-2010^‡^	0.55 (0.50-0.61)	0.57 (0.51-0.63)	1.74 (1.11-2.73)	1.54 (0.96-2.48)	0.52 (0.41-0.66)	0.52 (0.41-0.67)	0.54 (0.48-0.60)	0.56 (0.50-0.63)
CS acute 1999-2010^‡^	0.79 (0.73-0.84)	0.62 (0.58-0.67)	0.73 (0.42-1.27)	0.47 (0.27-0.83)	0.83 (0.72-1.07)	0.64 (0.55-0.75)	0.78 (0.72-0.84)	0.62 (0.57-0.67)
GA < 28 weeks	1.93 (1.57-2.37)	1.61 (1.31-2.00)	4.86 (1.81-13.01)	4.44 (1.66-11.92)	2.65 (1.78-3.93)	2.23 (1.48-3.35)	1.71 (1.35-2.17)	1.42 (1.11-1.83)
GA < 32 weeks	1.61 (1.43-1.81)	1.37 (1.21-1.55)	2.46 (1.17-5.20)	2.25 (1.06-4.74)	2.25 (1.78-2.84)	1.90 (1.50-2.41)	1.46 (1.26-1.66)	1.24 (1.07-1.43)
GA < 37 weeks	1.33 (1.27-1.40)	1.14 (1.08-1.20)	2.57 (1.94-3.42)	2.15 (1.60-2.87)	1.53 (1.38-1.70)	1.28 (1.15-1.42)	1.26 (1.20-1.33)	1.09 (1.03-1.15)
GA 37 – 41 weeks	0.91 (0.88-0.95)	1.03 (0.99 -1.06)	0.62 (0.49-0.79)	0.71 (0.55-0.90)	0.83 (0.77-0.89)	0.93 (0.86-1.01)	0.94 (0.91-0.98)	1.06 (1.02-1.10)
GA ≥42 weeks	0.92 (0.88-0.97)	0.85 (0.81-0.89)	0.87 (0.60-1.27)	0.84 (0.57-1.22)	0.96 (0.87-1.07)	0.91 (0.81-1.01)	0.91 (0.87-0.96)	0.84 (0.80-0.89)
Epidural analgesia^¥^	1.76 (1.72-1.81)	1.08 (1.05-1.11)	1.80 (1.49-2.18)	1.00 (0.82-1.22)	1.91 (1.81-2.02)	1.10 (1.03-1.16)	1.73 (1.68-1.78)	1.08 (1.05-1.11)
Perineal rupture grade 1-2^¥^	0.60 (0.58-0.62)	0.52 (0.50-0.54)	0.66 (0.51-0.86)	0.48 (0.37-0.64)	0.56 (0.52-0.61)	0.47 (0.43-0.52)	0.61 (0.58-0.63)	0.53 (0.51-0.55)
Perineal rupture grade 3-4^¥^	0.57 (0.51-0.64)	0.43 (0.38-0.49)	0.52 (0.19-1.39)	0.34 (0.13-0.91)	0.62 (0.47-0.81)	0.45 (0.34-0.59)	0.56 (0.49-0.64)	0.43 (0.37-0.49)
Preeclampsia	1.12 (1.03-1,23)	0.94 (0.86-1.03)	1.64 (0.93-2.91)	1.23 (0.67-2.25)	1.06 (0.86-1.31)	0.92 (0.74-1.14)	1.13 (1.02-1.24)	0.94 (0.85-1.04)
Abruptio placentae	1.31 (1.11-1.55)	1.19 (0.99-1.41)	0.57 (0.08-4.05)	0.59 (0.08-4.17)	1.46 (1.01-2.10)	1.34 (0.93-1.94)	1.29 (1.07-1.56)	1.17 (0.96-1.41)
Placenta praevia	0.34 (0.21-0.55)	0.37 (0.23-0.59)	2.53 (0.63-10.15)	2.72 (0.68-10.93)	0.11 (0.02-0.79)	0.12 (0.02-0.83)	0.35 (0.21-0.60)	0.38 (0.22-0.64)
PPH > 1000 ml (VD)	0.73 (0.67-0.79)	0.70 (0.64-0.76)	0.70 (0.37-1.31)	0.61 (0.33-1.15)	0.76 (0.63-0.91)	0.71 (0.60-0.85)	0.72 (0.66-0.79)	0.70 (0.64-0.76)
PPH > 1000 ml (CS)	0.93 (0.63-1.36)	0.98 (0.66-1.44)	N/A	N/A	1.74 (0.90-3.38)	1.71 (0.88-3.33)	0.77 (0.48-1.23)	0.75 (0.47-1.20)

Figures denote odds ratios and 95% confidence intervals. Reference group: Maternal age 20-30 years.

CS = Caesarean section; GA = gestational age at delivery; N/A = not applicable; PPH = postpartum haemorrhage; VD = vaginal delivery.

^†^ Adjusted for BMI and smoking habits at first antenatal care visit, parity and year of delivery. CS and preeclampsia also adjusted for gestational age.

^‡^ Caesarean section was subdivided into elective and acute CS from 1999.

¥ Epidural analgesia and perineal rupture among vaginal delivered women.

**Table 5:** Neonatal outcome data from singleton births to teenage women in the period 1992-2010

	**Age groups**							
	**All teenagers**		**< 16 years**		**16-17 years**		**18-19 years**	
**Characteristic**	**Crude**	**Adjusted**^**†**^	**Crude**	**Adjusted**^**†**^	**Crude**	**Adjusted**^**†**^	**Crude**	**Adjusted**^**†**^
Fetal distress	0.98 (0.81-1.20)	0.70 (0.57-0.85)	N/A	N/A	0.79 (0.47-1.32)	0.59 (0.36-0.99)	1.05 (0.85-1.30)	0.73 (0.59-0.91)
Aspiration of meconium	0.61 (0.42-0.90)	0.48 (0.33-0.72)	N/A	N/A	0.26 (0.06-1.03)	0.20 (0.05-0.82)	0.71 (0.47-1.06)	0.56 (0.37-0.84)
Shoulder dystocia	0.58 (0.46-0.74)	0.54 (0.42-0.69)	0.54 (0.08-3.84)	0.47 (0.07-3.38)	0.66 (0.39-1.12)	0.63 (0.37-1.07)	0.56 (0.43-0.74)	0.52 (0.40-0.69)
Stillbirth	1.09 (0.88-1.35)	1.07 (0.86-1.33)	0.77 (0.11-5.50)	0.82 (0.12-5.84)	1.50 (0.98-2.28)	1.50 (0.98-2.29)	1.01 (0.79-1.29)	0.98 (0.76-1.25)
SGA	1.58 (1.48-1.68)	1.07 (0.99-1.14)	1.82 (1.15-2.88)	1.30 (0.82-2.06)	1.49 (1.29-1.74)	0.96 (0.82-1.11)	1.60 (1.49-1.71)	1.09 (1.01-1.17)
LGA	0.56 (0.52-0.61)	0.85 (0.78-0.93)	0.20 (0.06-0.61)	0.32 (0.10-0.99)	0.51 (0.41-0.63)	0.83 (0.67-1.03)	0.58 (0.53-0.64)	0.86 (0.78-0.95)
Apgar score at 5 minutes < 7	1.17 (1.05-1.30)	1.00 (0.90-1.12)	1.64 (0.82-3.31)	1.27 (0.60-2.68)	1.22 (0.96-1.55)	1.06 (0.83-1.36)	1.15 (1.02-1.29)	0.99 (0.87-1.11)

Figures denote odds ratios and 95% confidence intervals. Reference group: Maternal age 20-30 years.

LGA = Large for gestational age; N/A = not applicable; SGA = Small for gestational age.

^†^ Adjusted for BMI and smoking habits at first antenatal care visit, parity and year of delivery.

Concerning the neonatal outcomes the neonates of the teenagers had significantly lower birth weight than the neonates of the adult women (ANCOVA; F_(3, 904286)_ = 16.160, p < 0.0001) although the differences in adjusted mean weight between all teenagers and the reference group was small, less than 30 gram (Table 3). The neonates were also less likely to show fetal distress and meconium aspiration in spite of a similar occurrence of low Apgar score at 5 minutes. Furthermore the neonates of the teenagers were less likely to be LGA but occurrence of SGA was similar with that of the adult women.

## Discussion

This large population based study showed that the rate of adolescent deliveries in Sweden has declined substantially during the last 35 years. The mode of delivery differed significantly between teenagers and adult women with significantly more normal vaginal deliveries and fewer CS among the teenagers. The teenagers were also less prone to perineal damage and PPH > 1000 ml. However, the teenagers ran a greater risk of adverse neonatal outcomes in terms of premature and very premature (<28 weeks of gestational age) delivery whereas fetal distress and meconium aspiration were seen significantly less often.

### Strengths and limitations

The strength of this study is the large data set registered prospectively representing an entire developed country. The MBR covers over 99% of all births taking place in Sweden and the prevalence of missing data in the MBR is low and the data have been validated and can therefore be considered to be reliable [20]. While this study was based on a fairly large dataset, it has to be considered that the data are from deliveries in Sweden alone. Sweden probably has one of the best organized systems in the world for providing perinatal care. It should therefore be emphasised that generalisation of the results to facilities outside the geographic and demographic area with different health systems and socio-demographic characteristics is uncertain. Generalization of the results should therefore be done with care. It is important to emphasize that teenage pregnancy can be associated with many more kinds of complications in other regions of the world. Furthermore we did not adjust for ethnicity and socioeconomic factors such as for instance geographical area, educational level or mean income of parents, which can be potential confounding factors when evaluating population-based biometric data. Data concerning ethnicity and socioeconomic status are not available in the MBR. Maternal anaemia during pregnancy could also be a confounder but this variable is not included in the MBR variables. However, maternal anaemia during pregnancy is uncommon in Sweden. At the antenatal visits all women have check-up of haemoglobin levels in early pregnancy (gestational week 10–13) as well as in gestational week 29. Supplemental iron and vitamins were offered routinely to all pregnant women in Sweden until 2008. The recommendation of iron supplementation has since then been based on serum ferritin levels which are measured routinely in early pregnancy. Some factors such as, for instance, parity, BMI and smoking habits are associated with being a teenager but are also associated with adverse perinatal outcomes. We therefore present both crude and adjusted OR’s in order to avoid an over adjustment in the interpretation of the results. To demonstrate possible causality between the different outcomes included in the analysis and maternal age a great number of putative intermediaries such as fetal size, oxytocin infusion and previous CS would have been considered, but determining causality was not the purpose of this study. Instead the effort was to evaluate outcomes in the teenage group of women overall. This approach may help clinicians interpreting the data.

### Interpretation

Although the annual number of deliveries varied in the different age groups the adolescent birth rate has almost universally declined with the exception of the years of “*all time high*” annual incidence of deliveries in Sweden (>100,000) during 1987 – 1994 when it increased slightly. This increase was mainly attributed to an increase in the number of deliveries by adolescents age 18 and 19 years. In spite of an increase in birth incidence during 2000 – 2010 the adolescent birth rate has still been declining. However, the oldest adolescents (19 years of age) have shown a moderate increase in birth rate. By also taking into consideration the information about the rate of adolescent births among adolescents showing an identical decline in birth rate we conclude that this strongly indicates that the Swedish strategies for family planning of adolescents have been successful in achieving the goal of reducing adolescent births and in particular reducing them among the very young adolescents. Interestingly we found that the percentage of teenagers that became multiparous among the teenagers who delivered during the study period was almost constant at a level of 7-10%. In the light of the decrease in adolescent deliveries overall the finding of a constant percentage of multiparous teenagers may indicate that implementation of preventive measures recommended and provided by the society concerning adolescent parenthood is wide spread in all socioeconomic groups.

The CS and instrumental delivery rates and perineal damage were significantly lower among adolescents compared with adult women. The reasons for these differences remain unclear. The very young age group showed a slightly different obstetric outcome in certain aspects. It is however important to emphasise the low number of individuals in this group and the small number of events. The findings should therefore be interpreted with great precaution. A low rate of instrumental deliveries among adolescents has almost unanimously been shown in several reports from developed as well as developing countries [4-6,10,11]. Whether this phenomenon depends on differences in handling the delivery, inherent or cultural behavioural, domestic or social attitudes among delivery staff has not been investigated. It cannot be ruled out that midwives and obstetricians may want to avoid obstetric interventions in pregnant teenagers to a greater extent than in older women because of concerns about future obstetric potential. It has previously been suggested that the biological factors could make the uterus and the genital tract of young women more favourable for accomplishing a normal delivery [22]. The fact that adolescents in our study in addition had a lower risk of perineal damage, PPH and placenta previa could support this biological explanation. However, all these issues need further investigation. Now that certain adverse outcomes have been eliminated in the teenage group a critical question in the future will be if we can extrapolate the care of adolescents during delivery to adult women?

In accordance with the findings of other authors our results showed that prematurity is a more common feature in adolescent pregnancies [1,3,5,7,10,23]. Olausson et al. found that the increased risk of preterm birth among adolescents was not influenced by possible confounders, including socioeconomic factors, suggesting that young age may be a biologic risk factor for preterm birth [7]. Our results seem to support this assumption but do not exclude other reasons.

Even though we found a significant association between birth weight and low maternal age this association seems to lack clinical relevance since the difference in adjusted mean weight between the groups was less than 30 gram. This interpretation is further supported by the fact that we failed to find an association between SGA and low maternal age as otherwise has been shown in other studies [1,3,5,23]. This discrepancy between our result and other reports might be explained by differences in socioeconomic conditions of populations, wealth, social and health care, indicating the effects of the high standards set for providing antenatal care in Sweden. In Sweden all women have access to the same antenatal and obstetric care and the care is completely free of charge for all women living in Sweden. The pregnant population is homogeneous, and poverty and malnutrition are practically non-existent among pregnant women [7,24]. Furthermore since the vast majority of pregnant women attend to the antenatal care program attendance is not related to maternal parity or age [25].

## Conclusion

This study demonstrates that the rate of adolescent births has decreased in Sweden. The risks for obstetric and neonatal complications for adolescents are lower than for women age 20–30 except for the risk of prematurity. It seems that other adverse neonatal outcomes among adolescents have been substantially eradicated in Sweden, most likely because of the high quality of antenatal, delivery and neonatal care and social welfare provided to all. The frequency of the modes of delivery differs between teenagers and adult women. This finding merits further investigations and raises the critical question: why aren’t adult women treated obstetrically in the same way as adolescents? We find it essential to continue the multi-disciplinary work in the society to further prevent adolescent parenthood and improve support for those adolescents who become parents.

## Abbreviations

ANCOVA: Analysis of covariance; BMI: Body mass index; CI: Confidence interval; CS: Caesarean section; ICD: International statistical classification of diseases and related health problems; LGA: Large for gestational age; SD: Standard deviation; MBR: Medical birth register; OR: Odds ratio; SGA: Small for gestational age.

## Competing interests

None of the authors has any conflict of interest to declare.

## Authors’ contributions

The study was planned and conducted by PK, MB and RBT, Data was analysed by all three. All authors contributed to the interpretation of the results, the elaboration of the manuscript and approval of the final version.

## Pre-publication history

The pre-publication history for this paper can be accessed here:

http://www.biomedcentral.com/1471-2393/13/204/prepub
